# GTN057, a komaroviquinone derivative, induced myeloma cells' death in vivo and inhibited c‐MET tyrosine kinase

**DOI:** 10.1002/cam4.5691

**Published:** 2023-02-24

**Authors:** Mikio Okayama, Kota Fujimori, Mariko Sato, Koichi Samata, Koki Kurita, Hiromu Sugiyama, Yutaka Suto, Genji Iwasaki, Taketo Yamada, Fumiyuki Kiuchi, Daiju Ichikawa, Maiko Matsushita, Maki Hirao, Hisako Kunieda, Kohei Yamazaki, Yutaka Hattori

**Affiliations:** ^1^ Division of Clinical Physiology and Therapeutics Keio University Faculty of Pharmacy Tokyo Japan; ^2^ Division of Hematology, Department of Internal Medicine Keio University School of Medicine Tokyo Japan; ^3^ Faculty of Pharmacy Takasaki University of Health and Welfare Takasaki Japan; ^4^ Department of Pathology Saitama Medical University Saitama Japan; ^5^ Division of Natural Medicines Keio University Faculty of Pharmacy Tokyo Japan; ^6^ Department of Hematology, Tokyo Saiseikai Central Hospital Tokyo Japan

**Keywords:** hepatocyte growth factor, komaroviquinone, multiple myeloma, natural product, tyrosine kinase inhibition

## Abstract

**Objective:**

Despite the development of newly developed drugs, most multiple myeloma (MM) patients with high‐risk cytogenetic abnormalities such as t(4;14) or del17p relapse at anin early stage of their clinical course. We previously reported that a natural product,komaroviquinone (KQN), isolated from the perennial semi‐shrub *Dracocephalum komarovi*, i.e., komaroviquinone (KQN) and its derivative GTN024 induced the apoptosis of MM cells by producing reactive oxygen species (ROS), but both exhibited significant hematological toxicity. Aim of this study is to clarify anti‐tumor activity, safety and pharmacokinetics of GTN057, an optimization compound of KQN in vivo.

**Methods:**

ICR/SCID xenograft model of KMS11, a t(4;14) translocation‐positive MM cell line, was used for in vivo study. Mice pharmacokinetics of GTN057 and the degradation products were analyzed by LC‐MS/MS.

**Results:**

Herein, our in vitro experiments revealed that GTN057 is much less toxic to normal hematopoietic cells, induced the apoptosis of both MM cell lines andpatient samples, including those with high‐risk cytogenetic changes. A xenograft model of a high‐risk MM cell line demonstrated that GTN057 significantly delayed the tumor growth with no apparent hematological or systemic toxicities in vivo. The pathological examination of GTN057‐treated tumors in vivoshowed revealed apoptosis of MM cells and anti‐angiogenesis. In addition to the production of ROS, GTN057 inhibited the downstream signaling of c‐MET, a receptor tyrosine kinase a receptor forand hepatocyte growth factor (HGF) receptor. Thus, GTN057 is less toxic and is able tomay be a candidate drug for treating MM patients, via multifunctional mechanisms. We have also extensively studied the pharmacologyical analysis of GTN057. The metabolites of GTN057, (e.g.,such as GTN054), may also have anti‐tumorantitumor activity.

**Conclusion:**

Natural products or and their derivatives can could be good sources of antineoplastic drugs even for high‐risk cancer.

## Graphical abstract

GTN057, a komaroviquinone derivative, induced myeloma cell death in vivo. No significant hematological or systemic toxicity was observed in vivo. The pharmacokinetic and pharmacological actions of GTN057 are also studied.

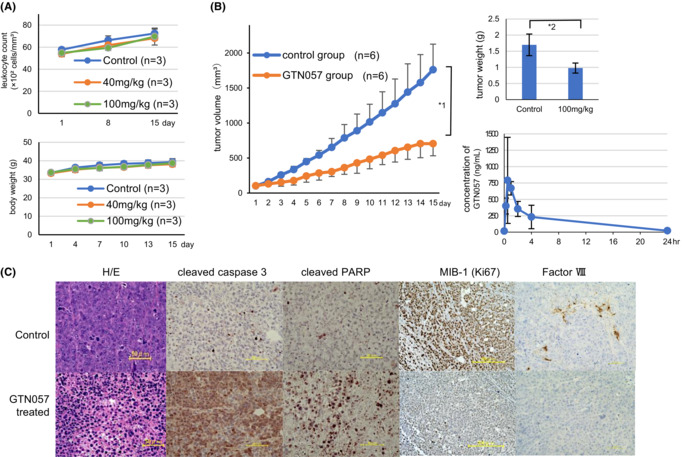

## 1 INTRODUCTION

As a hematopoietic tumor that is most common among elderly individuals,^1^ multiple myeloma (MM) causes characteristic clinical symptoms: progressive anemia, hypercalcemia, lytic bone lesion, renal insufficiency, and occasionally amyloid light‐chain (AL) amyloidosis. The prognoses of MM patients began to improve significantly in the early 2000s with the use of newly developed drugs^2^ including proteasome inhibitors,^3^, ^4^ immunomodulatory drugs (IMiDs),^5^ histone‐deacetylase inhibitor,^6^ and anti‐SLAMF7 (signaling lymphocytic activation molecule family member 7), and anti‐CD38 antibodies.^7^, ^8^ Proteasome inhibitors and IMiDs in particular have played a fundamental role in the treatment of MM and have significantly improved MM patients' prognoses.^9^

A number of MM patients whose MM cells have shown chromosomal abnormalities, for example, *t*(4;14), *t*(14;16), del17p, or 1q21 amplification, achieved significantly shorter survival regardless of the use of the above‐mentioned newly developed drugs and were thus categorized as having “high‐risk MM.”^10^, ^11^ The Revised International staging system for multiple myeloma describes high‐risk cytogenetic abnormalities as independent risk factors for poor prognosis.^12^ Since MM occurs mostly in elderly individuals, the side effects of new drugs occasionally impede the continuation of treatment. The further development of new drugs that are less toxic and more effective for high‐risk MM in clinical practice is greatly desired.

Komaroviquinone (KQN) is a natural product extracted from *Dracocephalum komarovii*, a perennial semi‐shrub native to central Asia. KQN shows anti‐protozoal activities against the organism that causes Chagas disease, that is, *Trypanosoma cruzi*.^13^ KQN killed *T. cruzi* via its reduction by TcOYE (*T. cruzi* old yellow enzyme) and its production of reactive oxygen species (ROS).^14^ Several new anti‐neoplastic drugs were isolated from natural products, especially those with anti‐protozoal activity,^15^ and we thus constructed a chemical library for KQN derivatives and screened the derivatives for the inhibition of the growth of MM cells that have high‐risk cytogenetic alterations.^16^, ^17^ By conducting a structure–activity relationship study, we found that the hydroquinone moiety is important for the potent antitumor effects of KQN derivatives.^17^ We observed that GTN024, a benzoquinone derivative of KQN, had high antitumor activities in MM cells. GTN024 also induced apoptosis in MM cells in vivo by producing ROS.^18^ However, GTN024 exhibited significant hematological toxicity in vitro, which hampers the clinical applications of this compound.^17^ To identify less‐toxic compounds, we continued our screening of the KQN‐derivatives library and observed that the KQN derivative GTN057 has an anti‐myeloma effect with negligible hematological toxicity in a colony assay (compound 18 in ref. (17)). Thus, GTN057 is a compound that merits further pharmacological study in vivo.

We speculated that GTN057 would have antitumor activities in MM cells in vivo. We conducted the present investigation to test this hypothesis, and we also investigated the anti‐MM mechanisms, safety, and pharmacokinetics of GTN057 in mice.

## 2 MATERIALS AND METHODS

### 2.1 Cells

Prof. Takemi Otsuki (Kawasaki Medical School, Kurashiki, Japan) kindly provided the cell lines KMM1, KMS11, KMS21, KMS26, KMS27, KMS28, and KMS34 from Japanese patients with MM.^19^ The MUM24 cells were obtained from a thalidomide‐resistant MM patient, and the authentication was confirmed by an STR (short tandem repeat)‐PCR (polymerase chain reaction) analysis (Japan National Institute of Biomedical Innovation, Osaka).^20^ All of the cell lines were cultured in RPMI1640 medium (Sigma‐Aldrich) containing 10% FBS (fetal bovine serum) (Gibco, Life Technologies) and 1% penicillin–streptomycin (Pen‐Strep, Gibco). Chromosomal abnormalities such as del(17p13.1) and *t*(4;14) were examined by a FISH (fluorescence in situ hybridization) analysis (LSI Medience, Tokyo). We have confirmed that del(17p13.1) is positive in the cell lines MUM24, KMS26, KMS28, KMS34, KMM1, and KMS11.^20^ In addition, *t*(4;14) translocation was observed in the MUM24, KMS26, KMS28, KMS34, and KMS11 cells^19^, ^20^ (see also Intini et al., Br J Haematol. 2004;126:437–9). The cell line A549 was obtained from the JCRB (Japanese Cancer Research Resources Bank) in 2002 and cultured in Eagle's MEM medium with 10% FBS and 1% penicillin–streptomycin (Gibco).

### 2.2 Reagents

**FIGURE 1:**
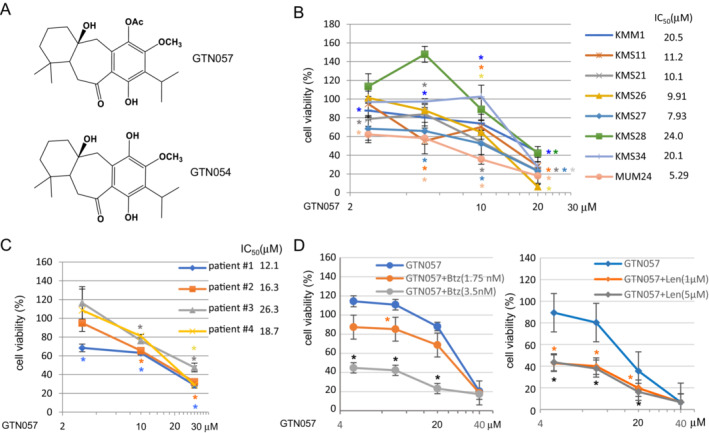
GTN057 induced the cell death of MM cells in vitro. (A) The chemical structure of GTN057 and the putative degradation product, GTN054. (B) GTN057 induced the cell death of the MM cell lines. The cells were incubated with GTN057 for 48 h, and the number of viable cells was counted by trypan blue exclusion test. Bars: mean ± SD (*n* = 3). *p < 0.05 versus control. (C) Growth‐inhibitory effects of GTN057 on CD138‐positive cells obtained from MM patients' bone marrow. Cells were treated with GTN057. The viability was evaluated by an MTT assay. Bars: mean ± SD (*n* = 3). *p < 0.05 versus control. (D) Combination treatment of GTN057 with bortezomib (Btz) or lenalidomide (Len), two major standard myeloma drugs, in MUM24 cells. The viability was evaluated by an MTT assay. Bars: mean ± SD (*n* = 3). *p < 0.05 versus GTN057 alone.

GTN057 was synthesized as described.^13^, ^16^, ^17^ Briefly, GTN054 was synthesized from komaroviquinone by palladium catalyzed hydrogenation (Figure 1A). The acetylation of GTN054 by acetic anhydride in the presence of triethylamine gave GTN057. The structure of GTN057 was revealed by ^1^H NMR (nuclear magnetic resonance), ^13^C NMR, IR (infrared) spectrometry, and mass spectrometry (see ref. 17 for details). For the in vitro investigation, phosphate‐buffered saline (PBS) containing 1% Tween® 80 (Otsuka Pharmaceuticals, Tokyo) and 10% dimethylsulfoxide (DMSO) was used as a solvent for GT057 dilution. For the in vivo administration, GTN057 was diluted in 1% Tween® 80% + 10% sodium dodecyl sulfate (SDS) in saline.

### 2.3 Patient samples

Mononuclear cells were obtained from the bone marrow aspirates of MM patients treated at Tokyo Saiseikai Central Hospital, by centrifugation with Lymphoprep™ (Axis‐Shield). The mononuclear cells were then labeled with CD138 MicroBeads from Miltenyi Biotec (Bergisch Gladbach). The labeled CD138‐positive cells were magnetically purified using MACS Columns (Miltenyi Biotec).

### 2.4 Growth inhibition of MM cells by GTN057

For the trypan blue exclusion assay, 2 × 10^5^ cells/mL of each MM cell line were incubated with various concentrations of GTN057 (0–20 μM). The cells were cultured in triplicate plates, and viable cells were evaluated by a trypan blue exclusion assay (Trypan Blue Stain 0.4%, Gibco) using an automatic cell counter (TC20™, Bio‐Rad).

For the 3‐[4,5]‐2,5‐diphenyltetrazolium bromide (MTT) assay (Roche Diagnostics), 6 × 10^4^ cells/mL of CD138‐positive bone marrow cells were cultured in 96‐well plates in triplicate with various concentrations of GTN057 for 48 h. The cell viability was examined by MTT dye absorbance in accord with the manufacturer's manual. We obtained the IC_50_ values by drawing approximate straight lines in semilogarithmic growth curves, using Microsoft Excel.

### 2.5 Reactive oxygen species (ROS) production

First, 2 × 10^5^ cells/mL of MUM24 cells were incubated with or without 6 mM NAC (N‐acetyl cysteine; Sigma‐Aldrich) or 3 mM GSH (glutathione; Sigma‐Aldrich) for 2 h. Next, 20 μM GTN057 was added and cultured for an additional 72 h. Cells were incubated with 1 μM CM‐H_2_DCFDA (i.e., 5‐(and‐6)‐chloromethyl‐2′, 7′‐dichlorodihydrofluorescein diacetate) for 30 min. For the evaluation of ROS production, the cells were stained with CM‐H_2_DCFDA and analyzed using a FACS (fluorescence‐activated cell sorting) system (LSR II, BD Biosciences). For the assessment of growth recovery by NAC or GSH, 2 × 10^5^ cells/mL of MUM24 cells were also treated with 20 μM GTN057 in the presence or absence of 6 mM NAC or 3 mM GSH as described above. We conducted a trypan blue exclusion test to determine the numbers of viable cells.

### 2.6 Western blotting

MM cells (4 × 10^5^ cells/mL) were incubated with 0–20 μM GTN057 for 12 h. The cells were then co‐cultured with 0–50 ng/mL of hepatocyte growth factor (HGF) (PeproTech, London) or 0–20 ng/mL of fibroblast growth factor (FGF)‐2 (PeproTech) with 1 unit/mL of heparin in RPMI containing 4% dialyzed FCS (Gibco) for the KMS11 and KMS34 cells and 10% dialyzed FCS for the KMS21 cells for 15 min at RT (room temperature). A549 cells were co‐cultured with 0–50 ng/mL of epidermal growth factor (EGF) (PeproTech) in Eagle's MEM containing 4% dialyzed FCS for 15 min at RT. The cells were lysed in 1% NP‐40 buffer containing 50 mM Tris/HCl, pH 8.0, 1% NP‐40, 150 mM NaCl, 1 mM PMSF, 1 mM Na_3_VO_4_, 20 mM NaF, 2 mM Na_4_P_2_O_7_, and protease inhibitors (Complete Protease Inhibitor Mixture, Roche Diagnostics, Mannheim, Germany). After centrifugation of the lysates (15,000 rpm for 10 min), the supernatants were saved and stored at −80°C until use.

The protein concentrations were measured using a BCA Protein Assay Kit (Thermo Fisher Scientific). The lysates were diluted with 3 × Laemmli's sample buffer (0.25 M Tris/HCl, pH 6.8, 4% SDS, 0.006% bromophenol blue, and 6% 2‐mercaptoethanol) and boiled for 5 min. The lysates were then subjected to 10% SDS‐PAGE (sodium dodecyl sulfate‐polyacrylamide gel electrophoresis) and transferred to PVDF (polyvinylidene fluoride) membranes. Five‐percent skim milk was used to block the membranes overnight at 4°C, and the membranes were then subjected to Western blotting.

Antibodies against phospho‐STAT3 (Tyr705), phospho‐Akt (Ser473), p44/42 MAPK (Erk 1/2), phosphor‐p44/42 MAPK (Thr202/Tyr 204), and phospho‐c‐Met (Tyr1234/1235) (all from Cell Signaling Technology, Danvers, MA) or STAT3, Akt, c‐Met, and β‐actin (all from Santa Cruz Biotechnology, Santa Cruz, CA) or phospho‐c‐Met (Tyr1230/1234/1235) (Biosource, Camarillo, CA for KMS34 cells in Figure 3A) were each used at 1:1000 dilution. The second antigen‐antibodies, that is, HRP (horseradish peroxidase)‐conjugated anti‐rabbit or anti‐mouse Ig antibody, were each used at 1:5000 dilution. Details regarding the antibodies used for Western blotting are provided in Table S1. The Western blotting signals were detected by enhanced ECL chemiluminescence (Amersham). The signal intensity of each band was quantified densitometrically with ImageJ software (ver. 1.48, U.S. National Institutes of Health).

### 2.7 Animal experiments

This study was performed in strict compliance with Keio University's Institutional Guidelines on Animal Experimentation to minimize the animals' suffering. All animal experiments (including the toxicity assay, the in vivo tumor growth assay, and the pharmacokinetic study) were prospectively approved by Keio University's Institutional Animal Care and Use Committee (no. 12067‐(5)). All mice were kept in a specific pathogen‐free (SPF) space in which pathogen monitoring was carried out every 6 months. As humane endpoints in in vivo tumor growth assay, if the inoculated tumor size reached 3000 mm^3^, the mouse was sacrificed by isoflurane treatment or cervical dislocation. In our experiments including the toxicity assessment, in vivo tumor growth assay, or pharmacokinetic study, all mice were euthanized by isoflurane treatment or cervical dislocation. No mouse died of any other reason. Nine male 5‐week‐old ICR mice were used for the toxicity assessment. Twelve male 5‐week‐old ICR/SCID mice were used for the in vivo xenograft model and histopathologic investigation. The pharmacokinetic study was performed using three male 5‐week‐old ICR mice.

### 2.8 Toxicity assessment

For the toxicity assessment of GTN057 in vivo, we intraperitoneally (i.p.)‐injected male 5‐week‐old ICR mice (CLEA, Tokyo) with GTN057 (0, 50, or 100 mg/kg) dissolved in 1% Tween® 80 + 10% DMSO in saline on 2 consecutive days of every 3‐day period for 2 weeks. The mice were weighed every week. Peripheral blood was collected with a heparinized hematocrit tube (Terumo, Tokyo) from the tail vein of each mouse every week and was stained with Türk's solution (Merck). The numbers of leukocytes and neutrophils were counted using light microscopy.

### 2.9 In vivo tumor growth assay

For the in vivo tumor growth assay, we injected 3 × 10^7^ cells of KMS11 cells subcutaneously into the flank of male 5‐week‐old ICR/SCID mice. When the tumors reached 100 mm^3^, we injected (i.p.) 20 or 100 mg/kg of GTN057 solubilized in 1% Tween® 80 + 10% DMSO in saline twice every 3 days for 2 weeks. We measured the tumor volumes every day for 2 weeks as the length × width^2^ × 0.52.^21^

### 2.10 Histopathologic examination

Xenografts were excised and fixed with 10% formalin and embedded in paraffin. Five‐μm sliced sections were used for hematoxylin and eosin (H&E) staining. For the immunohistochemical evaluation, we used anti‐human cleaved poly(ADP‐ribose), polymerase (PARP), polyclonal antibody (pAb) (Cell Signaling Technology Japan, Tokyo), anti‐human Ki‐67 monoclonal antibody (mAb) (clone MIB‐1) (Dako Japan, Tokyo), anti‐human cleaved caspase‐3 (Asp175), pAb (Cell Signaling Technology Japan), and anti‐human Factor VIII mAb (Atlas Antibodies). Detailed information about these antibodies is provided in Table S1.

### 2.11 Pharmacokinetic study

For the pharmacokinetic study, 0 or 100 mg/kg of GTN057 (1% Tween® 80 + 10% DMSO in saline) was injected (i.p.) into male 5‐week‐old ICR mice. Peripheral blood samples were taken using heparinized hematocrit tubes from the tail vein of each mouse at 0, 15, and 30 min and 1, 2, 4, and 24 h. The blood samples were applied to centrifugation (1100 × *g*, 15 min at 4°C) and we then collected the supernatant plasma. CH_2_CN (Wako) and MeOH (Kanto, Tokyo) for LC–MS/MS (liquid chromatography–tandem mass spectrometry) measurements were mixed in a 1:1 ratio, and verapamil hydrochloride (an internal standard substance) was dissolved to the final concentration of 120 ng/mL to prepare a deproteinizing agent. We diluted the blood samples four times with this deproteinizing agent. After 15 min on ice, each deproteinized plasma sample was centrifuged (9100 × *g*, 10 min, 4°C). The supernatants were collected and used for the LC–MS/MS analyses.

### 2.12 LC–MS/MS analysis

All compounds including GTN057 were analyzed with a mass spectrometer (AB SCIEX) coupled to an HPLC (high‐performance liquid chromatography) system (Shimadzu). The peptides were separated with the use of an Atlantis® dC18, 3‐μm, 100 Å column (Waters). The solvent composition was 15% for solution A (100 mM CH_3_COONH_4_) and 85% for solution B (CH_3_OH). The HPLC measurement settings were as follows. Constant flow rate: 0.2 mL/min, column temperature: 40°C. The MS settings were: spray voltage, 5500 V; turbo heater temperature, 300°C.

### 2.13 Statistical analyses

**FIGURE 2:**
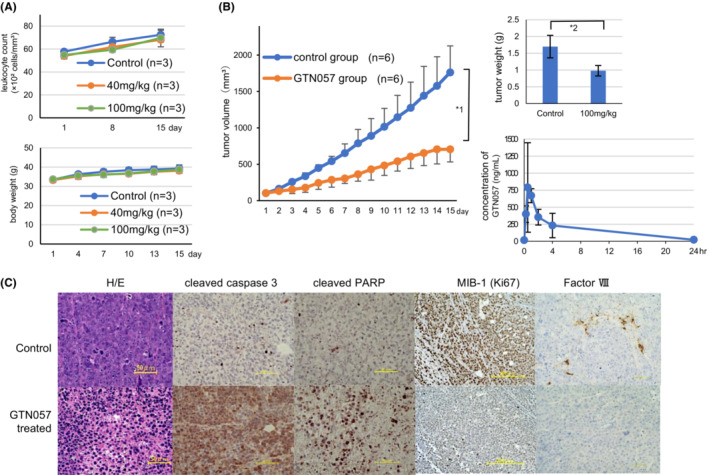
Growth inhibition and toxicity of GTN057 in the mouse xenograft model. (A) 40 or 100 mg/kg of GTN057 was subcutaneously injected into ICR mice on 2 consecutive days of each 3‐day period until day 14. Leukocyte and neutrophil counts in the peripheral blood collected from tail veins were evaluated by staining with Türk's solution. Bars: mean ± SD (*n* = 3). (B) In the xenograft model using deletion 17 and *t*(4;14)‐positive KMS11 cells, 100 mg/kg of GTN057 was injected (i.p.) on 2 consecutive days of each 3‐day period until day 14. Bars: mean ± SD (*n* = 6). *1 *p* = 0.00016, *2 *p* = 0.0015 at day 15 (control vs. GTN057). The results of the pharmacokinetic study of GTN057 at 0, 5, 15, 30, 45 and 60 min and 2, 4, 8, 12, and 24 h after a single peritoneal injection are shown. GTN057 (m/z 405) peaks were detected by LC–MS/MS, and the concentrations were calculated. (C) Histopathological findings of a representative xenografted tumor of a mouse treated with GTN057. Excised xenografts after treatment with GTN057 or control were stained with H&E. Immunohistochemical examinations were also conducted using anti‐cleaved caspase‐3, anti‐cleaved PARP, anti‐MIB‐1 (Ki67), and anti‐Factor VIII antibodies.

**FIGURE 3:**
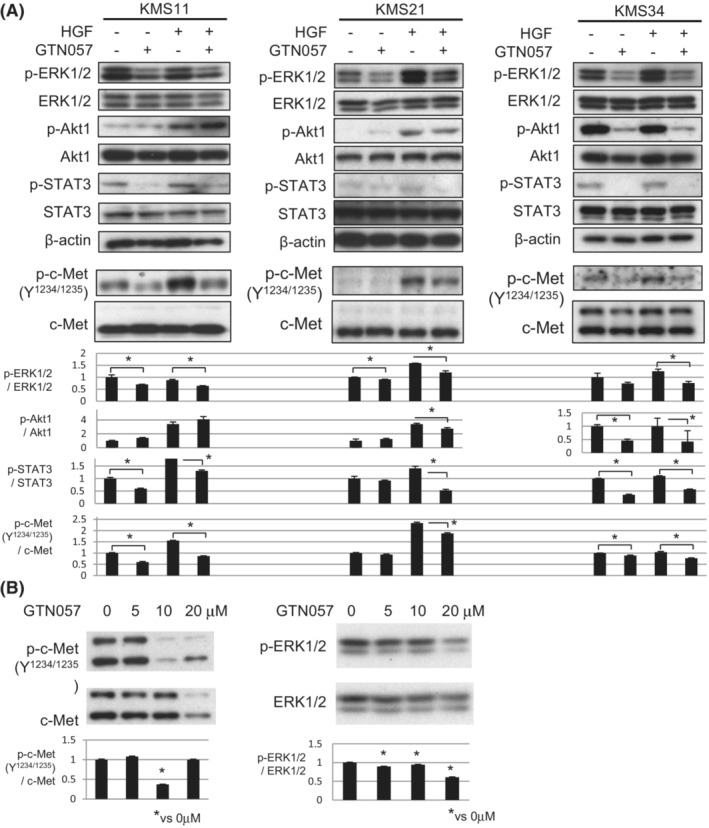
The inhibition of HGF/c‐MET signaling by GTN057. (A) KMS11, KMS21, or KMS34 cells were treated with 50 ng/mL of HGF in the presence/absence of 20 μM GTN057. The phosphorylations of ERK1/2, AKT1, STAT3, and c‐MET/HGF receptor were examined by immunoblot analyses. (B) The dose‐dependent inhibition of c‐MET and the downstream signaling by GTN057 in KMS34 cells. The results of the densitometric analysis of the activation of c‐MET and ERK1/2, a signal transducer, are also shown (A, B). *p < 0.05, Student's *t*‐test.

We used an unpaired Student's *t*‐test with a two‐tailed distribution to determine the significance of differences in the growth inhibition of MM cells in vitro (Figure 1B–D, Figure S1B), in the in vivo tumor growth assay (Figure 2A,B), Western blots (Figure 3), and pharmacokinetics (Figure 2B, Figure S4B) with Microsoft Excel. Probability (*p*)‐values <0.05 were accepted as significant.

## 3 RESULTS

### 3.1 GTN057 inhibited the MM cells' proliferation

We investigated whether GTN057 would inhibit the growth of the five MM cell lines in vitro by conducting a trypan blue exclusion assay. In each of the cell lines examined, GTN057 inhibited the cells' proliferation in a dose‐dependent manner. The IC_50_ (half‐maximal inhibitory concentration) values calculated for the MUM24, KMS11, KMS21, KMS26, and KMS27 cells were 5.29, 11.2, 10.1, 9.91, and 7.93 μM, respectively (Figure 1B). In the KMM1, KMS28, and KMS34 cells, 50% growth inhibition (IC_50_) was observed at 20.5, 24.0, and 20.1 μM, respectively. Chromosome 17 was deleted in MUM24, KMM1, KMS11, KMS26, KMS28, and KMS34 cells, and *t*(4;14) translocation was detected in the MUM24, KMS11, KMS26, KMS28, and KMS34 cells (see the Materials and Methods section). Thus, GTN057 was able to kill multiple lines of myeloma cells, including the MM cell lines with high‐risk cytogenetic changes.

We conducted an MTT assay to examine whether GTN057 can inhibit the growth of CD138^+^ cells obtained from four MM patients. As shown in Figure 1C, GTN057 inhibited the growth of the cells from each of the patients. The IC_50_ values were 12.1 μM in Patient 1, 16.3 μM in Patient 2, 26.3 μM in Patient 3, and 18.7 μM in Patient 4. Four patients showed high‐risk cytogenetic changes: Patient 1 had 1q21 amplification; Patient 2 had 1q21 amplification, *t*(4;14) and *t*(14;16); Patient 3 had 1q21 amplification and del 17p; and Patient 4 showed 1q21 amplification and *t*(11;14) and a complex karyotype change.

We also examined the combination treatment of GTN057 with bortezomib and lenalidomide, two major standard myeloma drugs. As shown in Figure 1D, GTN057 exerted an additive anti‐myeloma effect with these two drugs.

### 3.2 The toxicity of GTN057 in the mouse model

For the assessment of the toxicity of GTN057 in the mouse model, 40 or 100 mg/kg of GTN057 was injected (i.p.) to ICR mice on 2 consecutive days of each 3‐day period for 2 weeks (e.g., on days 1, 2, 4, 5, 7, 8, 10, 11, 13, and 14). When the mice were treated with either dose of GTN057, we observed neither a loss of body weight nor leukocytopenia (Figure 2A). We thus considered GTN057 to be a highly safe compound, with 100 mg/kg or higher as the maximal tolerated dose in the animal experiments at the injection schedule of 2 days of every 3‐day period.

### 3.3 The anti‐multiple myeloma activity of GTN057 in vivo

For the determination of the anti‐multiple myeloma activities of GTN057 in vivo, we injected (i.p.) 100 mg/kg per day of GTN057 into KMS11‐xenografted SCID mice. As illustrated in Figure 2B, GTN057 significantly inhibited the MM cells' growth in the KMS11‐xenografted mice compared to the controls. After 2 weeks of treatment, the average tumor volume of the GTN057‐treated mice was significantly smaller than that of the control mice (1.76 ± 0.36 cm^3^ vs. 0.71 ± 0.17 cm^3^, *p* = 0.00016). The average tumor weight was also significantly lower in the GTN057‐treated mice (1.70 ± 0.34 g vs. 0.98 ± 0.16 g, *p* = 0.0015). We performed LC–MS/MS to determine the plasma concentrations of GTN057 after a single i.p. injection (100 mg/kg) in ICR mice. The C_max_ was 790.1 ng/mL (1.95 μM), the t_max_ was 30 min, and the plasma disappearance half‐time was 110 min (Figure 2B). At 24 h post‐injection, the injected GTN057 had almost completely disappeared from the blood.

In our pathological examinations of the GTN057‐treated xenografts, the H&E staining revealed a chromatin aggregation in the GTN057‐treated tumor cells' nuclei (Figure 2C). In addition, the immunohistochemical examinations revealed significantly increased numbers of cleaved‐caspase‐3‐positive and PARP‐positive cells in the GTN057‐treated tumors. By contrast, GTN057 treatment weakened the staining for the cell proliferation marker MIB‐1/Ki67 (Figure 2C). These pathology results suggested that GTN057 induced apoptosis and growth inhibition in MM cells in vivo. We also observed that the number of Factor VIII‐positive epithelial vessel cells was decreased in the GTN057‐treated xenografts, suggesting that GTN057 could inhibit tumor angiogenesis.

### 3.4 GTN057's production of ROS

KQN was reported to show anti‐protozoal activity via the production of ROS, mediated by protozoal old yellow enzyme.^13^, ^14^ In our previous study, KQN and its derivative GTN024 produced ROS in MM cells, which was suspected to be the antitumor mechanism of this derivative.^17^, ^18^ We thus investigated whether GTN057 can also produce ROS and induce ROS‐dependent MM cell death in the present study, and we observed that GTN057 also produced ROS in MM cells (Figure S1). However, the MM cells' growth inhibition and apoptosis induced by GTN057 were not significantly abrogated by co‐culturing with anti‐oxidants (Figure S1). We thus speculated that an additional anti‐myeloma mechanism of GTN057 exists.

### 3.5 The effects of GTN057 on the HGF signaling pathway

To clarify the mechanisms that underlie the functions of GTN057, we attempted to elucidate GTN057's effect on intracellular growth signaling. We focused on the phosphorylations of ERK1/2, STAT3, and Akt1, which are known to be activated by various growth factors and cytokines. Since we previously reported the growth‐promoting effect of HGF in MM cells, in the present study we investigated whether GTN057 regulated the phosphorylations of ERK1/2, STAT3, and Akt1 in MM cells when they were stimulated by HGF or other growth factors.^21^ As shown in Figure 3, the phosphorylations of ERK1/2, STAT3, and Akt1 were enhanced when KMS11, KMS21, and KMS34 cells were treated with HGF. The addition of GTN057 diminished the degrees of the phosphorylation of ERK1/2, STAT3, and Akt1.

In addition, anti‐phospho c‐MET antibody, which reacts with ERK1/2 and STAT3‐binding site y^1234^ y^1235^, successfully detected the phosphorylation of c‐MET/HGF receptor induced by HGF treatment. Co‐culture with GTN057 and HGF abolished the phosphorylation at y^1234^ y^1235^ of the c‐MET protein (Figure 3A). The basal phosphorylation level of c‐MET and the downstream signal transducers varied in myeloma cells. One of the reasons for this variation is the autocrine activation of c‐MET in KMS11 and KMS34 cells, as our previous observation showed that these two cell lines produce HGF.^21^ In the present study we also performed a Western blot analysis using various concentrations of GTN057 in KMS34 cells to examine the inhibition of c‐MET and the downstream signaling (Figure 3B). The 50% inhibition of the phosphorylations of c‐MET and ERK1/2 by GTN057 was observed at 35.9 μM and 21.3 μM, respectively (Figure 3B), and these results are comparable with the IC_50_ value for growth inhibition, 20.1 μM (Figure 1B). However, GTN057 did not clearly change the ERK1/2, STAT3, or AKT1 phosphorylation levels in KMS11 MM cells, in which FGFR1 and FGFR3 were expressed, when the cells were stimulated by FGF‐2 (Figure S2). In the A549 lung cancer cell line (in which EGF receptors are overexpressed), the phosphorylation levels of ERK1/2, STAT3, and AKT1 were not altered by GTN057 when these cell lines were activated by epidermal growth factor (EGF) stimulation (Figure S2). GTN057 thus preferentially inhibited HGF signaling.

## 4 DISCUSSION

Several anti‐neoplastic drugs have been isolated and developed from products that are present in plants. For example, a derivative of camptothecin, that is, irinotecan, inhibits topoisomerase I and was isolated from *Camptotheca acuminata*, a deciduous tree.^22^ Etoposide (VP16) also inhibits topoisomerase II and is a derivative of podophyllotoxin, which is an ingredient of the perennial plant *Podophyllum peltatum*.^23^ Paclitaxel, which depolymerizes and consequently stabilizes microtubules, was isolated from the tree *Taxus brevifolia*.^24^ Vincristine and paclitaxel inhibit tubulin polymerization and were isolated from the flowering plants *Vinca rosea* (*Catharunthus roseus*; niche niche sou) and *Taxus brevifolia*, respectively.^25^, ^26^ Doxorubicin and mitomycin, both of which target DNA, were derived from Streptomyces species.^27^, ^28^

In recently developed cancer treatments, molecular targeted drugs are preferred due to their lower toxicity to normal cells. Some molecular targeted drugs have also been developed from products found in nature. For example, trichostatin A, an extract from *Streptomyces hygroscopicus*, contributed to the development of vorinostat, which inhibits histone deacetylase activity.^29^, ^30^ ATRA (all‐trans retinoic acid), which is used for the differentiation induction therapy of APL (acute promyelocytic leukemia), is a biologically active form of vitamin A.^31^ Natural products are thus expected to continue to be a source of lead compounds for novel antineoplastic drugs. In the present study, we chose a novel natural compound, KQN, as a lead compound that could act against high‐risk MM.

KQN and KQN‐related compounds were successfully synthesized by Suto and colleagues.^13^, ^17^ We reported that GTN024 effectively induced myeloma cell‐death; however, the toxicity of GTN024 to normal hematopoietic cells was of serious concern.^18^ It was reported that GTN057 did not inhibit the colony formation of bone marrow cells from mice.^17^ As described in the Results section, when mice were injected with 100 mg/kg of GTN057 on 2 days of each 3‐day period over a 2‐week period, no significant neutropenia or systemic toxicity was observed, indicating the low toxicity of this compound.

Our present findings demonstrated that GTN057 significantly inhibited the proliferation of both the five MM cell lines and the CD138‐positive cells obtained from the bone marrow of patients with MM. Our mouse xenograft model showed that significant anti‐MM effects occurred following the injection schedule of 2 days of each 3‐day period without significant toxicities. The histopathological examination demonstrated that GTN057 treatment resulted in the apoptosis of xenografted KMS11 cells, which have *t*(4;14) translocation and *TP53* gene deletion (Figure 2). Thus, GTN057 is a promising candidate molecule for overcoming high‐risk MM.

It has also been shown that *c‐MET* gene knock‐down in myeloma cells increased the cells' susceptibility to bortezomib and doxorubicin.^32^, ^33^ Thus, sensitization to existing anti‐myeloma drugs might also be an anti‐myeloma effect of GTN057. Despite the significant anti‐myeloma effect of GTN057 in the present xenograft model, the C_max_ value of GTN057 was lower than the IC_50_ values for the MM cells (Figure 2) when mice were injected with GTN057. One of the possible reasons for this result is that the degradation products of GTN057 also decrease myeloma cells' proliferation in vitro.^17^ Our preliminary data demonstrated that GTN057 (*m/z* 405 in Figure S3) was immediately dehydrated (*m/z* 387) or deacetylated (*m/z* 363) in plasma into several compounds (Figure S3). A deacetylated product of GTN057, that is, GTN054 (*m/z* 363) (Figure 1A), compound 17 in ref. (17) showed potent antiproliferative activity in our previous study, in which the IC_50_ of MUM24 cells was 6.13 μM.^17^ By conducting an ion scan analysis, we also detected and analyzed putative peaks of GTN057 and the metabolites in LC–MS/MS (Figure S3). The time course changes of the plasma concentrations of GTN054 in mice are illustrated in Figure S4. We speculate that further dehydrated compounds of GTN054 (e.g., *m/z* 345 in Figure S3) may also have antitumor activity. We recognized that GTN057 per se possesses anti‐myeloma activity and also works as a prodrug. Thus, GTN057 exerted significant antiproliferative activity with a low C_max_ and a short half‐life in the mouse xenograft model.

The production of ROS, which is a major anti‐myeloma activity of GTN024,^18^ is not the only antiproliferative mechanism of GTN057; we observed herein that GTN057 inhibited HGF receptor/c‐Met in addition to the downstream signaling mediated by ERK1/2, STAT3, and AKT1. The exact molecular mechanisms underlying the inhibition of c‐Met by GTN057 are not yet known. To reveal the direct binding of GTN057 to c‐Met, biophysical experiments such as a three‐dimensional structure analysis are needed for future study. The activation of the HGF/c‐Met pathway has been elucidated in various types of cancer including gastric cancer, non‐small lung cancer, and hepatic cancer.^34^ The HGF/c‐Met signal pathway is also known to have roles in angiogenesis and the invasion and metastasis of tumors, and it is thought to be a promising treatment target.^35^, ^36^ Investigations of multiple myeloma have shown that (i) both HGF and c‐Met signaling are involved in the growth of MM cells, and (ii) the plasma concentration of HGF is related to disease progression and/or prognosis.^21^, ^37^, ^38^ It was also suggested that an HGF/c‐Met signal is involved in bone marrow angiogenesis and disease activity in MM.^21^, ^39^

In our present histopathological observations using the mouse model, the number of Factor VIII‐positive vascular endothelial cells in the GTN057‐treated tumors decreased, suggesting that GTN057 could inhibit tumor angiogenesis. Our earlier study showed that an antagonist against HGF, that is, NK4, also inhibited HGF/c‐MET signaling and anti‐angiogenic activity in a mouse MM xenograft model, indicating the growth dependency of myeloma cells on HGF/c‐MET.^21^ It was also reported that HGF inhibited bone morphogenetic protein (BMP), which is an important factor for the differentiation of osteoblasts.^40^ This suggested that GTN057 might improve bone lesions in MM patients by inhibiting the HGF signaling pathway.

In conclusion, we screened a KQN‐derivative library considering the structure‐antitumor activity relationship, and we identified GTN057, which is less toxic to normal tissues and induced MM cells' apoptosis in vivo via multifunctional mechanisms including tyrosine kinase inhibition, ROS production, and anti‐angiogenesis.

## AUTHOR CONTRIBUTIONS

**Mikio Okayama:** Investigation (equal). **Kota Fujimori:** Investigation (equal); methodology (equal); writing – original draft (supporting). **Mariko Sato:** Investigation (equal); methodology (equal). **Koichi Samata:** Investigation (equal). **Koki Kurita:** Investigation (equal); methodology (equal). **Hiromu Sugiyama:** Investigation (equal); methodology (equal). **Yutaka Suto:** Conceptualization (equal); data curation (equal); resources (lead); writing – original draft (equal). **Genji Iwasaki:** Resources (equal). **Taketo Yamada:** Investigation (equal); methodology (equal). **Fumiyuki Kiuchi:** Conceptualization (equal); data curation (equal); writing – original draft (equal). **Daiju Ichikawa:** Software (equal). **Maiko Matsushita:** Writing – original draft (equal). **Maki Hirao:** Resources (equal). **Hisako Kunieda:** Resources (equal). **Kohei Yamazaki:** Resources (equal). **Yutaka Hattori:** Conceptualization (lead); data curation (lead); formal analysis (lead); funding acquisition (lead); investigation (equal); methodology (lead); project administration (lead); resources (supporting); software (equal); supervision (lead); validation (lead); visualization (lead); writing – original draft (lead).

## CONFLICT OF INTEREST STATEMENT

The following potential conflicts of interests exist: Y.H. has received research grants from Takeda Pharmaceutical Co., MSD, Astellas Pharma, Daiichi Sankyo Co., Pfizer, Ono Pharmaceutical Co., and AbbVie GK.

## ETHICAL APPROVAL STATEMENT

The Ethics Committee of Tokyo Saiseikai Central Hospital (No. 28–66) and the Faculty of Pharmacy, Keio University (No. 220518–5) approved the use of bone marrow samples from myeloma patients. The study conforms to the provisions of the Declaration of Helsinki. All of the patients provided written informed consent for their samples to be used.

## Supporting information


Figure S1.

Figure S2.

Figure S3.

Figure S4.

Table S1.

## Data Availability

The data obtained in this study are available from the corresponding author upon reasonable request.
