# Supplementary figures and images for: GTN057, a komaroviquinone derivative, induced myeloma cells' death in vivo and inhibited c‐MET tyrosine kinase
Source: Cancer Med. 2023 Feb 24;12(8):9749–59. doi: 10.1002/cam4.5691 (PMC10166914; doi:10.1002/cam4.5691)

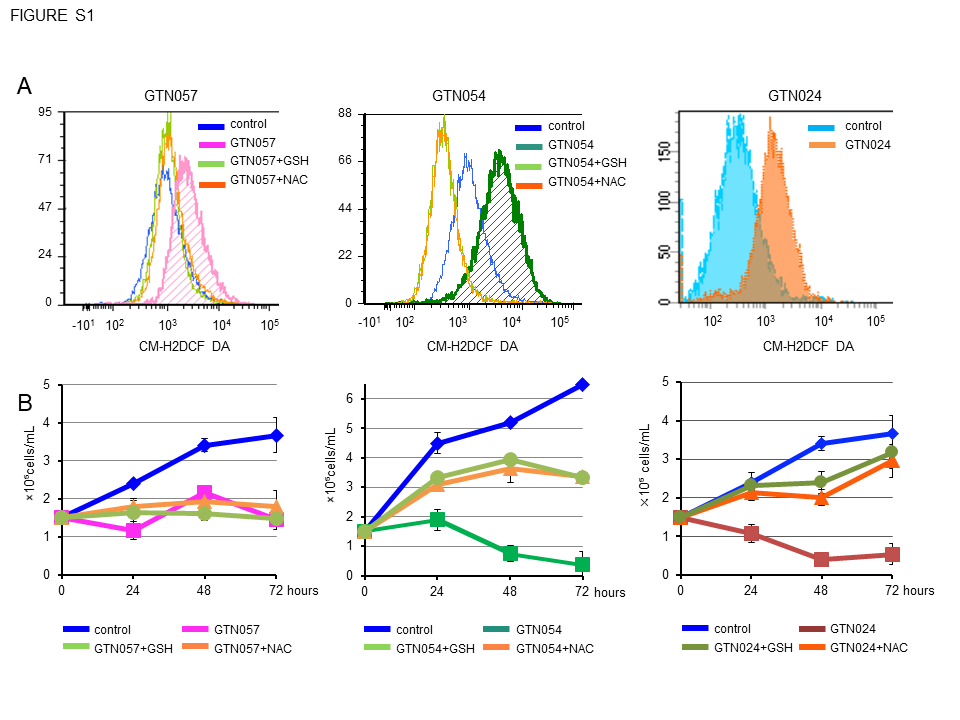

Supplement: Supplementary file 1 — Figure S1. [file CAM4-12-9749-s004.tif]

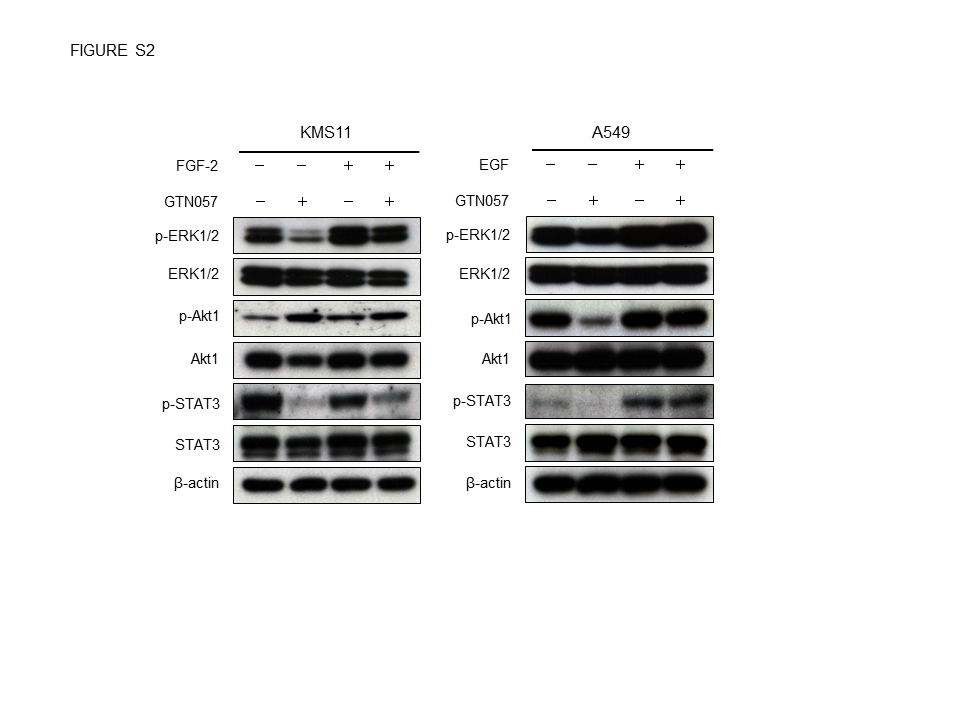

Supplement: Supplementary file 2 — Figure S2. [file CAM4-12-9749-s001.tif]

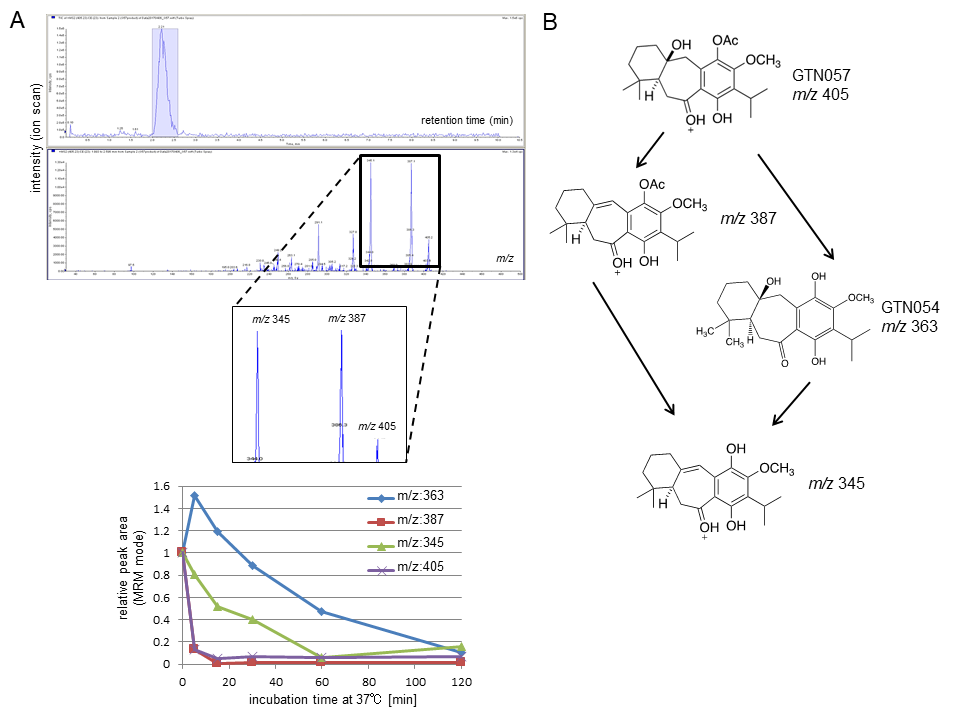

Supplement: Supplementary file 3 — Figure S3. [file CAM4-12-9749-s005.tif]

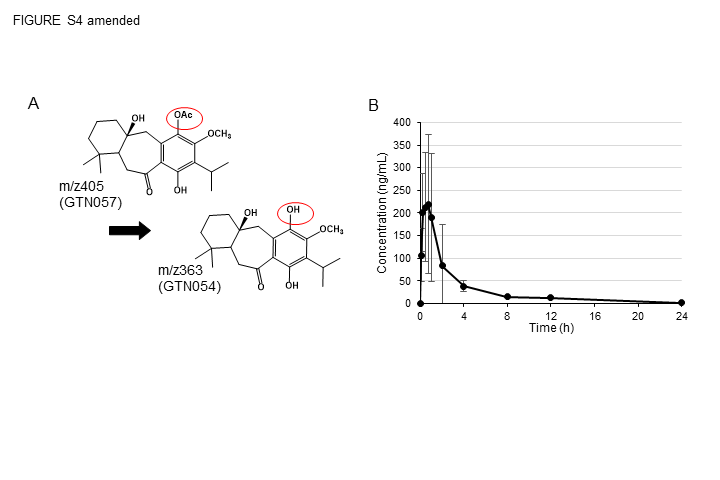

Supplement: Supplementary file 4 — Figure S4. [file CAM4-12-9749-s002.tif]
