# Supplementary material for: GTN057, a komaroviquinone derivative, induced myeloma cells' death in vivo and inhibited c‐MET tyrosine kinase
Source: Cancer Med. 2023 Feb 24;12(8):9749–59. doi: 10.1002/cam4.5691 (PMC10166914; doi:10.1002/cam4.5691)
Supplement: Supplementary file 5 — Table S1. [file CAM4-12-9749-s003.doc]

| **Table S1 Information of antibodies used in western blot analyses and immunohistochemistry** | | |  |  |  |  |
| --- | --- | --- | --- | --- | --- | --- |
| antibodies | catalog number | company | clonality | immune animal | dilution | purpose |
| anti-phospho-STAT3 (Tyr705) | #9131 | Cell Signaling Technology | polyclonal | rabbit | 1:1,000 | western blot |
| anti-phospho-AKT1 (Ser473) | #9271 | Cell Signaling Technology | polyclonal | rabbit | 1:1,000 | western blot |
| anti-phospho-p44/42 MAPK (Thr202/Tyr204) | #9101 | Cell Signaling Technology | polyclonal | rabbit | 1:1,000 | western blot |
| anti-p44/42 MAPK (Erk1/2) | #9102 | Cell Signaling Technology | polyclonal | rabbit | 1:1,000 | western blot |
| anti-phospho-c-Met (Tyr1234/1235) | #44-888G | Biosource | polyclonal | rabbit | 1:1,000 | western blot |
| anti-phospho-c-Met (Tyr1234/1235) | #3077 | Cell Signaling Technology | monoclonal | rabbit | 1:1,000 | western blot |
| anti-STAT3 (C-20) | #sc-482 | Santa Cruz Biotechnology | polyclonal | rabbit | 1:1,000 | western blot |
| anti-AKT1 (C-20) | #sc-1618 | Santa Cruz Biotechnology | polyclonal | goat | 1:1,000 | western blot |
| anti-c-Met (C-12) | #sc-10 | Santa Cruz Biotechnology | polyclonal | rabbit | 1:1,000 | western blot |
| anti-β-actin (H-196) | #sc-7210 | Santa Cruz Biotechnology | polyclonal | rabbit | 1:1,000 | western blot |
| HRP-conjugated anti-rabbit immunoglobulin | P0448 | DAKO | polyclonal | goat | 1:5,000 | western blot |
| HRP-conjugated anti-mouse immunoglobulin | P0260 | DAKO | polyclonal | rabbit | 1:5,000 | western blot |
| anti-goat immunoglobulin | sc-2033 | Santa Cruz Biotechnology | polyclonal | donkey | 1;2,000 | western blot |
| anti-human cleaved PARP | #9541S | Cell Signaling Technology | polyclonal | rabbit | 1;500 | immunohistochemistry |
| anti-human cleaved caspase-3 (Asp175) | #9661 | Cell Signaling Technology | polyclonal | rabbit | 1;300 | immunohistochemistry |
| anti-human Ki-67 antibody (clone MIB-1) | M7240 | DAKO | monclonal | mouse | 1;200 | immunohistochemistry |
| anti-human Factor VIII | M0616 | DAKO | monclonal | mouse | 1;100 | immunohistochemistry |
| ImmPRESS® HRP-conjugated anti-rabbit IgG Polymer Detection Kit | MP-7401 | VECTOR | polyclonal | horse | ready-to-use | immunohistochemistry |
| ImmPRESS® HRP-conjugated anti-mouse IgG Polymer Detection Kit | MP-7402 | VECTOR | polyclonal | horse | ready-to-use | immunohistochemistry |
